# Case Report: Late Complication of a Dry Socket Treatment

**DOI:** 10.1155/2010/479306

**Published:** 2011-01-04

**Authors:** Ramón Manuel Alemán Navas, María Guadalupe Martínez Mendoza

**Affiliations:** ^1^Department of Oral and Maxillofacial Surgery, Zacamil National Hospital, Evangelical University of El Salvador, San Salvador 00106-8000, El Salvador; ^2^Faculty of dentistry, Latin American University, 03100 Mexico City, Mexico

## Abstract

Dry socket is often treated in dentistry with intra-alveolar dressings; the use of them remains controversial and has been related to some side effects such as neuritis, foreign body reactions, and myospherulosis. We present a case of an intra-alveolar dressing (zinc-oxide eugenol paste) that mimicked a trigeminal neuralgia for 3 years and caused a right maxillary chronic osteomyelitis and foreign body reaction in a zone corresponding to the alveolus of the maxillary first molar. This long-term complication was successfully managed by complete removal of the foreign body and curettage of the affected area.

## 1. Introduction

Dry socket is a common complication following a tooth extraction, with a peak incidence in the 40–45 year-old age group. It has an incidence of 1%–4% for all routine dental extractions and is more frequent in female patients [1, 2]. It is a self-limiting disease that often will take 5–10 days to disappear, even without treatment. The treatment of this disease has commonly been divided into two groups: the nondressing and dressing interventions. The use of dressing interventions is controversial because no scientific studies have been carried out that specifically to investigate the incidence of potential side effects and tissue damage arising from the placement of them [3]. These dressings according to their active principle can be classified into antimicrobial dressings, soothing dressings, or dressings with local anesthetics [4]. One of the most common dressings reported in the literature is zinc oxide and eugenol, often mixed into a semisolid consistency [1, 5]. Local complications have been described after the placement of intra-alveolar dressings; some of them are neuritis [6], foreign body reactions [7, 8], and Myospherulosis [9, 10]. This paper presents a case of a late complication related to a dry socket dressing that mimicked a trigeminal neuralgia during 3 years and caused a chronic osteomyelitis with foreign body reaction.

## 2. Case Report

A 45-year-old female was referred to the oral and maxillofacial department of Zacamil's national hospital; the chief complaint was a right trigeminal neuralgia that could not be managed by conservative treatment. It all began 3 years before when the right maxillary first molar was extracted. Four days after the extraction her dentist diagnosed a dry socket, which was treated with an intra-alveolar dressing consisting of zinc-oxide eugenol paste; this medication was placed directly into the alveolus without any other transport vehicle. The patient experienced relief of pain and never went back with her dentist so paste remained inside the alveolus (this information was obtained directly from the medical files of the dentist that treated the patient). 

Several weeks later, the patient presented a right maxillary pain. She visited different dentists to find the cure to her pain, and during a period of two years third molar, second molar, first premolar, and second premolar of the right maxillary side were extracted. Hemifacial pain persisted, and the patient was referred to the neurologist who confused by the symptoms treated the patient as a trigeminal neuralgia. Carbamazepine was prescribed for about a year without pain relief; after this the neurologist sent the patient to our department.

The chief complaint was an intermittent right hemifacial pain, which was described as an ache with periods of intense shooting pain. A visual analog scale was used to measure it during the intermittent periods finding a severe pain. Physical examination revealed no trigger zones and clinical absence of teeth 1, 2, 3, 4, and 5. 

**Figure 1:**
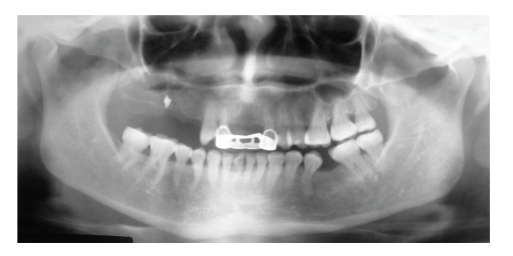
Panoramic X-ray. Notice the presence of a right maxillary radiopacity and the absence of teeth 1, 2, 3, 4, and 5, all with adequate bone healing except the 3 area.

**Figure 2:**
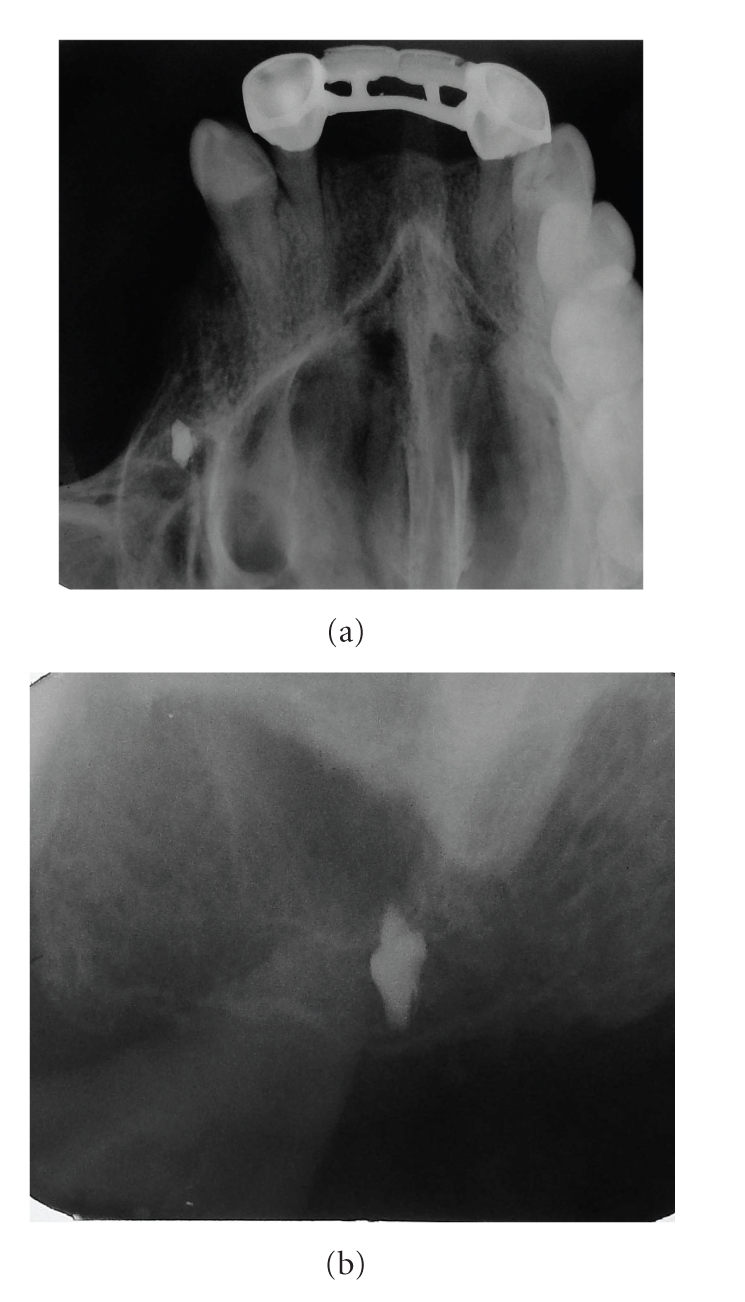
(a) Maxillary occlusal X-ray. (b) Periapical X-ray. Notice the closure of the foreign body to the right maxillary sinus, and also a nonhealed 3 alveolus.

**Figure 3:**
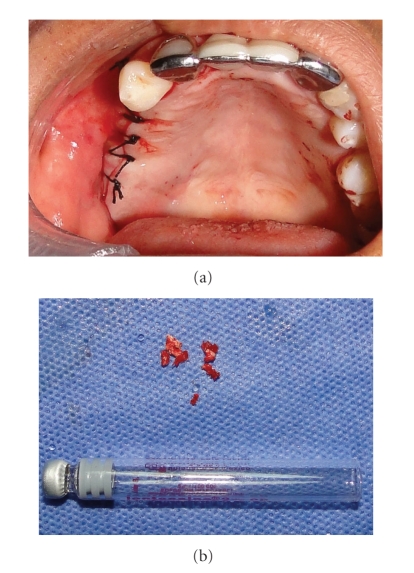
(a) Intraoral supracrestal approach, through which foreign body was removed and incision was sutured with silk 3-0. (b) Macroscopical view of the foreign body removed, which all together measured 0.7 × 0.5 × 0.2 cms.

**Figure 4:**
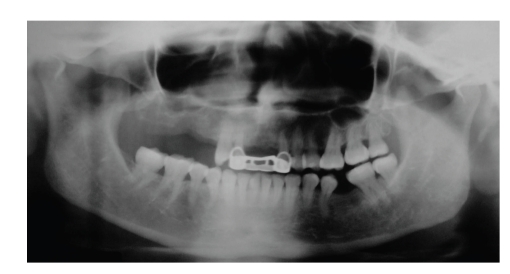
Panoramic X-ray shows a complete postoperative view without the foreign body.

**Figure 5:**
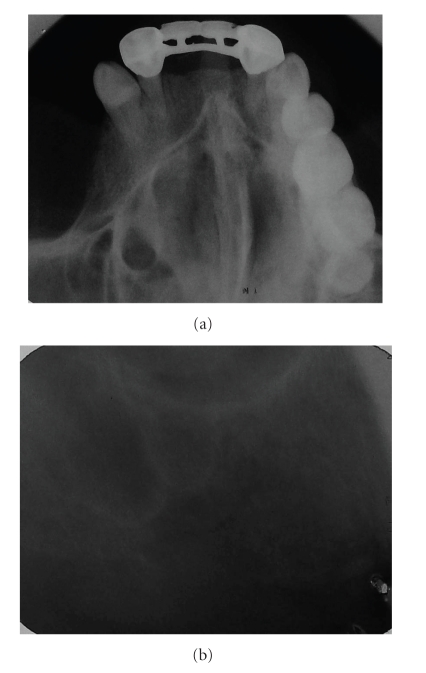
(a) Postoperative maxillary oclusal X-ray. (b) Postoperative periapical X-ray. Notice the complete removal of the right maxillary foreign body.

Panoramic, oclusal, and periapical X-rays were taken. A right maxillary foreign body was found in the position of a nonhealed alveolar bone of the maxillary first molar; the image was in close proximity to maxillary sinus (Figures 1 and 2). Due to the signs and symptoms of the patient, the foreign body was removed and curettage of the affected area was done; the findings during surgery were: a nonhealed alveolus, granulation tissue, free bone fragments, and a white solid foreign body that was in direct contact with maxillary sinus (Figure 3). All tissues were sent to the pathologist who reported a well-vascularized fibrous connective tissue, chronic inflammatory infiltrate, multinucleated giant cells and necrotic bone surrounded by bacterial forms. The final diagnosis was a chronic osteomyelitis with zones of foreign body reaction. Antibiotics and nonsteroidal anti-inflamatory drugs were used the week after the surgery. The rest of the postoperative care was managed in a conventional manner without any further complications. Postoperative X-rays showed adequate healing and complete removal of the foreign body (Figures 4 and 5).

After one week of surgery the patient experienced relief of pain. Visual analog scale was used during several months revealing no pain after foreign body was removed. Patient has been followed up for six months without any facial pain during this time.

## 3. Discussion

The number of secondary complications to the placement of dressings in the treatment of an established dry socket is ignored; most of the complications previously reported, myospherulosis, neuritis, and foreign body reaction, are related to intra-alveolar medication as a preventive methods and not as a treatment [4]. 

Bright et al. 1982 [10] and Belfiglio et al. in 1986 [9] described myospherulosis related to tetracycline in a petrolatum base, used as a preventive measure to avoid dry socket. Now is known that petroleum-based carriers interfere with wound healing by action of lipids on extravasated erythrocytes, producing myospherulosis. Because of this, nowadays the usage of petroleum-based carriers has been discouraged [1]. 

Zuniga and Leist in 1995 reported a topical tetracycline induced neuritis six months after routine removal of an unerupted mandibular third molar [6]. Moore and Brekke in 1990 reported a foreign-body giant cell reaction related to placement of tetracycline-treated polylactic acid [7]. Mainous in 1974 reported foreign body reaction after zinc oxide-eugenol packing in localized osteitis [8]. Bloomer in 2000 did an investigation of the prevention of alveolar osteitis by immediate placement of medicated packing but they used the medication for one week only and then they removed it, so they did not report complications in a long-term evaluation [11]. 

Oil of cloves is eugenol in its unrefined form, and it has been used for centuries as a toothache remedy. Chisholm described its mixture with zinc oxide in 1873 to form a plastic mass for therapeutic uses. It has sedative and anodyne effects as well as antibacterial properties. This mixture of eugenol with zinc oxide relies on a setting reaction between them which produces zinc eugenolato. Eugenolato is not stable in the presence of water, and readily undergoes hydrolysis with the release of free eugenol. Free eugenol can also be of detriment to human soft tissues. The type and extent of oral tissues reactions to eugenol vary but eugenol is generally cytotoxic at high concentrations and has an adverse effect on fibroblasts and osteoblast-like cells. Thus, at high concentrations, it produces necrosis and reduced healing. This effect is dose related and will potentially affect all patients [5, 12]. Eugenol is also neurotoxic, able to cause interruption of neural transmission. Kozam noted that eugenol at certain concentrations can extinguish impulse transmission of a nerve within 3 hours. Also transient paresthesias have been reported after the use of eugenol as an endodontic medication [13, 14]. Other treatment using a packing has been reported for the effective relief of alveolar osteitis pain which includes using iodoform gauze (NU Gauze, Johnson & Johnson Wound Management) coated with a mixture of three to five drops of obtundant, eugenol, with or without other ingredients, and packed into the anesthetized socket. This treatment should not be used if the patient is allergic to iodine [15]. In our case, the intra-alveolar zinc-oxide eugenol medication, caused bone necrosis, foreign body reaction, delayed alveolar healing, and hemifacial pain that was confused with a trigeminal neuralgia. Zinc-oxide eugenol probably caused a neurotoxic effect in the affected area. Symptoms of the patient confused dentists and neurologist, misleading them to the wrong diagnosis of a trigeminal neuralgia. 

This case reveals the need to do more long-term scientific investigations about the usage of intra-alveolar dressings as treatment for dry socket and not as prevention of it, in order to determine the safety of them and their potential side effects to our patients in long-term studies. It also reveals the importance of a thorough clinical and radiographic evaluation in patients with a suspected diagnosis of trigeminal neuralgia to discard local jaws affections that could confuse or mislead to a wrong diagnosis and treatment. Finally, providing patients with written postoperative instructions stating what was placed in the socket, how long it should stay in the socket, and when or if it should be removed, should not be overlooked by treating physician.
